# Mistakes in terminology cause false conclusions: Vitamin D does not increase the risk of dementia

**DOI:** 10.1111/acel.13722

**Published:** 2022-09-29

**Authors:** Reinhold Vieth

**Affiliations:** ^1^ Department of Laboratory Medicine and Pathobiology, Department of Nutritional Sciences, Faculty of Medicine University of Toronto Toronto Ontario Canada

**Keywords:** 1,25‐dihydroxy, 25‐hydroxyvitamin D, Alzheimer's disease, calcidiol, calcitriol, cognition, dementia, epidemiology, hormone, kidney disease, nutrition, vitamin D

## Abstract

There has been a progressive trend in recent years, to trivialize the terminology surrounding the molecules based on a secosteroid structure. The generic use of the term, “vitamin D," results in gross misrepresentations that confuse the use of a drug commonly used for patients with kidney failure, with the nutritional use of vitamin D. This commentary is a critique of one particularly bad example of that terminological trivialization. Authors may simply want to add impact to their findings when they refer to “vitamin D supplementation” when what they are reporting on is calcitriol. However, the consequences of this practice are to mislead all readers who do not go through the primary publication very carefully to understand the details behind sloppy terminology. Contrary to all the words written in the publication commented upon here, it offers no clinical evidence that vitamin D supplementation increases risk of Alzheimer's disease or dementia.

Any connection between dementia or Alzheimer's disease and something so basic to nutrition as vitamin D warrants serious attention. This commentary is a criticism of the authors, reviewers, and editors who were involved with the recent report in the journal, Cell Ageing, which claims that “vitamin D supplementation worsens Alzheimer's progression” (Lai et al., 2022). The publication is attention grabbing in the most egregious way. The only real consequence of the report by Lai et al is that it creates unnecessary worry and confusion for vulnerable people seeking information.

For the animal and cell‐culture aspects of the report by Lai et al, it was not unreasonable that the concentrations of vitamin D, calcidiol (25(OH)D) and calcitriol (1,25(OH)2D) used were many orders of magnitude beyond natural or physiological concentrations. High doses are often used for that kind of research to elicit the changes that justify their publication because journals rarely publish in vitro experiments that show negative results.

To translate their laboratory findings into the clinical context, Lai et al obtained patient data from the National Health Insurance Research Database in Taiwan. They investigated whether there was an association between dementia that was newly diagnosed after the year 2000, and a government‐paid prescription for calcitriol between the years 2000 and 2010. Indeed, they did find an association between being prescribed calcitriol and a greater risk of a diagnosis of dementia (Lai et al., 2022). Beyond that, Lai et al grossly equated any prescription to the drug, calcitriol, as being the same as the use of the nutrient, vitamin D. That misrepresentation happens throughout the publication: in the title, the abstract, the discussion, and the conclusion. The results section even states that (0.25 or 0.5 mcg) daily is the suggested optimal daily intake for this vitamin, which is a wild misrepresentation of the reality that dietary recommendations are closer to 15 mcg/day (600 IU/day) (Bouillon, 2017).

Lai et al. provide no information as to why calcitriol was prescribed to the people whose data they reported on. However, the medical indications for prescribing calcitriol are well known: “Calcitriol is used to treat and prevent low levels of calcium and bone disease in patients whose kidneys or parathyroid glands… are not working normally. It is also used to treat secondary hyperparathyroidism” (AHFS Patient Medication Information Database, 2016). Aside from the diminished glomerular filtration rate that defines kidney failure, the disease limits the kidney's ability to produce the hormone, calcitriol; therefore, hormone replacement with calcitriol is started. Unless physicians in Taiwan do not prescribe calcitriol properly, the patients using this drug are mainly people suffering from substantial kidney disease.

The logical conclusion of the epidemiological part of the work of Lai et al is that people with moderate to severe kidney disease are at greater than normal risk of an eventual diagnosis of dementia. This has been widely reported for many years, that kidney disease accelerates cognitive decline. That decline happens regardless of whether or not the patients have been prescribed calcitriol (Hailpern et al., 2007; Lu'o'ng & Nguyễn, 2011; Martens et al., 2017). The clinical portion of Lai et al is misleading; moreover, without realizing it, the authors' work is confirmatory of the relationship between kidney disease and dementia or cognitive decline.

It is essential that anyone involved in the stages of publishing about vitamin D needs to know the basic reality that the vitamin D obtained via the skin from sun exposure, from foods, or supplements is an inactive molecular precursor. Vitamin D is not a hormone. The thing that is measured as an index of vitamin D nutrition is serum 25(OH)D, which circulates in a physiological concentration range extending up to 200 nmol/L (80 ng/ml). Like the vitamin D that it comes from, 25(OH)D is not a hormone and it is essentially inactive. Lai et al provided no data and made no mention of the actual vitamin D nutritional status of the people in their study (Lai et al., 2022). Finally, the hormone, calcitriol (1,25(OH)2D), is produced by the kidney, it is not a “vitamin,” and it is extremely potent, its secretion is tightly regulated, and it circulates at minuscule concentrations of about 120 picomol/L (48 pg/ml). Calcitriol is particularly toxic, and unlike its precursors, it has a very narrow window of safety (Vieth, 2020).

To refer to vitamin D as a hormone is as absurd as referring to cholesterol as a steroid hormone. An inactive molecular precursor is not the same thing as a hormone. It is high time for everyone to stop the ridiculous practice of calling any molecule based on a classic secosteroid structure, "vitamin D."

## CONFLICT OF INTEREST

None.

## AUTHOR CONTRIBUTION

The author solely conceived, wrote and submitted the manuscript for publication.

## Data Availability

Data sharing is not applicable to this article as no new data were created or analyzed in this study.

## References

[acel13722-bib-0003] AHFS Patient Medication Information Database . (2016). “Calcitriol: MedlinePlus Drug Information” . https://medlineplus.gov/druginfo/meds/a682335.html

[acel13722-bib-0001] Bouillon, R. (2017). Comparative analysis of nutritional guidelines for vitamin D. Nature Reviews Endocrinology, 13(8), 466–479. 10.1038/nrendo.2017.31 28387318

[acel13722-bib-0002] Hailpern, S. M. , Melamed, M. L. , Cohen, H. W. , & Hostetter, T. H. (2007). Moderate chronic kidney disease and cognitive function in adults 20 to 59 years of age: Third National Health and Nutrition Examination Survey (NHANES III). Journal of the American Society of Nephrology: JASN, 18(7), 2205–2213. 10.1681/ASN.2006101165 17554148

[acel13722-bib-0004] Lai, R.‐H. , Hsu, C.‐C. , Yu, B.‐H. , Lo, Y.‐R. , Hsu, Y.‐Y. , Chen, M.‐H. , & Juang, J.‐L. (2022). Vitamin D supplementation worsens Alzheimer's progression: Animal model and human cohort studies. Aging Cell, e13670. 10.1111/acel.13670 35822270 PMC9381901

[acel13722-bib-0005] Lu'o'ng, K. V. Q. , & Nguyễn, L. T. H. (2011). The beneficial role of vitamin D in Alzheimer's disease. American Journal of Alzheimer's Disease and Other Dementias, 26(7), 511–520. 10.1177/1533317511429321 PMC10845314 22202127

[acel13722-bib-0006] Martens, R. J. H. , Kooman, J. P. , Stehouwer, C. D. A. , Dagnelie, P. C. , van der Kallen, C. J. H. , Koster, A. , Kroon, A. A. , Leunissen, K. M. L. , Nijpels, G. , van der Sande, F. M. , Schaper, N. C. , Sep, S. J. S. , van Boxtel, M. P. J. , Schram, M. T. , & Henry, R. M. A. (2017). Estimated GFR, albuminuria, and cognitive performance: The Maastricht study. American Journal of Kidney Diseases, 69(2), 179–191. 10.1053/j.ajkd.2016.04.017 27291486

[acel13722-bib-0007] Vieth, R. (2020). Vitamin D supplementation: Cholecalciferol, calcifediol, and calcitriol. European Journal of Clinical Nutrition, 74(11), 1493–1497. 10.1038/s41430-020-0697-1 32704098

